# T2‐Weighted intracranial vessel wall imaging at 7 Tesla using a DANTE‐prepared variable flip angle turbo spin echo readout (DANTE‐SPACE)

**DOI:** 10.1002/mrm.26152

**Published:** 2016-02-18

**Authors:** Olivia Viessmann, Linqing Li, Philip Benjamin, Peter Jezzard

**Affiliations:** ^1^ FMRIB Centre, Nuffield Department of Clinical NeurosciencesJohn Radcliffe HospitalOxfordOX3 9DUUK; ^2^ Molecular Imaging Branch, National Institute of Mental HealthNational Institutes of HealthBethesdaMaryland, USA; ^3^ Neurosciences Research Centre, St George's HospitalUniversity of LondonUK

**Keywords:** Vessel wall imaging, black‐blood imaging, stroke, plaque imaging, variable flip angle, DANTE, SPACE

## Abstract

**Purpose:**

To optimize intracranial vessel wall imaging (VWI) at 7T for sharp wall depiction and high boundary contrast.

**Methods:**

A variable flip angle turbo spin echo scheme (SPACE) was optimized for VWI. SPACE provides black‐blood contrast, but has less crushing effect on cerebrospinal fluid (CSF). However, a delay alternating with nutation for tailored excitation (DANTE) preparation suppresses the signal from slowly moving spins of a few mm per second. Therefore, we optimized a DANTE‐preparation module for 7T. Signal‐to‐noise ratio (SNR), contrast‐to‐noise ratio (CNR), and signal ratio for vessel wall, CSF, and lumen were calculated for SPACE and DANTE‐SPACE in 11 volunteers at the middle cerebral artery (MCA). An exemplar MCA stenosis patient was scanned with DANTE‐SPACE.

**Results:**

The 7T‐optimized SPACE sequence improved the vessel wall point‐spread function by 17%. The CNR between the wall and CSF was doubled (12.2 versus 5.6) for the DANTE‐SPACE scans compared with the unprepared SPACE. This increase was significant in the right hemisphere (*P* = 0.016), but not in the left (*P* = 0.090). The CNR between wall and lumen was halved, but remained at a high value (24.9 versus 56.5).

**Conclusion:**

The optimized SPACE sequence improves VWI at 7T. Additional DANTE preparation increases the contrast between the wall and CSF. Increased outer boundary contrast comes at the cost of reduced inner boundary contrast. Magn Reson Med 77:655–663, 2017. © 2016 The Authors Magnetic Resonance in Medicine published by Wiley Periodicals, Inc. on behalf of International Society for Magnetic Resonance in Medicine. This is an open access article under the terms of the Creative Commons Attribution License, which permits use, distribution and reproduction in any medium, provided the original work is properly cited.

## INTRODUCTION

Intracranial vessel‐wall imaging (VWI) is of interest in research into cerebrovascular diseases that implicate vessel wall pathology. Certain types of ischemic strokes, eg, lacunar infarcts, can result from blockages of the lenticulostriate arteries near their origins at the middle cerebral artery (MCA) 1, 2. Clinically established methods, such as computed tomography angiography, magnetic resonance angiography, and digital subtraction angiography detect only the lumen of the vessel, and early thickening of the wall is likely to be missed. VWI of the MCA could potentially allow detection of early pathology. The increase in signal‐to‐noise ratio (SNR) and spatial resolution at 7T would benefit VWI, provided that 7T‐specific challenges can be accounted for, such as the specific absorption rate (SAR) and blurring for long spin echo readout trains 3, 4. The two conditions that must be met by intracranial VWI are that 1 both blood and the cerebrospinal fluid (CSF) signal are sufficiently suppressed to resolve the inner and outer lumen, and 2 the wall signal is kept at an acceptable level to create contrast.

Variable flip angle sequences were initially developed to reduce SAR and blurring at higher field and have effectively replaced the classic 180 ° turbospin echo (TSE) readout at 7T 5, 6. Another feature of nonselective variable flip angle schemes is their black‐blood effect 7, 8. The scheme calculation requires tissue relaxation times $T_{1}$ and $T_{2}$. The vendor‐implemented constants are not optimal for vessel wall tissue at 7T. In this study we sought to optimize a variable flip angle sequence (sampling perfection with application optimized contrast using different flip angle evolution (SPACE) 9) to produce a sharp depiction of vessel wall. Although it effectively suppresses fastflowing blood spins, SPACE has a limited effect on slowly flowing CSF spins in the vicinity of the MCA. To accomplish the latter, we used the signal crushing capability of the delay alternating with nutation for tailored excitation (DANTE) preparation module, which has been shown to be sensitive to slow velocities in the order of mm per second 10 and has been used previously to suppress CSF in the spinal cord 11. Recently, a combination of DANTE and variable flip angle TSE has been used by Wang et al 12 and Xie et al 13 to improve VWI at 3T. We assessed the wall conspicuity of the default and optimized SPACE sequence by point‐spread function (PSF) analysis. We measured the SNR of the three compartments in the MCA: vessel wall, CSF, and lumen for both DANTE‐SPACE and SPACE sequences. Additionally, we calculated the signal ratio (SR) and contrast‐to‐noise ratio (CNR) between them.

## METHODS

### Sequence Diagram

**Figure 1:**
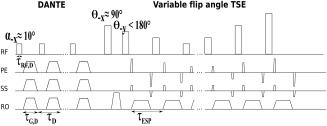
Sequence diagram of DANTE‐SPACE. The DANTE preparation module consists of a train of nonselective low flip angle pulses followed by strong gradients along all principal directions. The DANTE module is followed by a nonselective 90° pulse into the transverse plane and nonselective variable flip angles of less than 180°.

Figure 1 shows a schematic representation of the DANTE‐SPACE pulse sequence. The SPACE readout consists of an initial 90 ° tip angle into the transverse plane followed by a train of refocusing pulses of variable flip angles less than 180 °. The DANTE module is a train of short nonselective radiofrequency (RF) pulses, followed by strong gradients that introduce a high‐order phase‐spoiling effect on moving spin isochromats 10.

### SPACE Optimization

**Figure 2:**
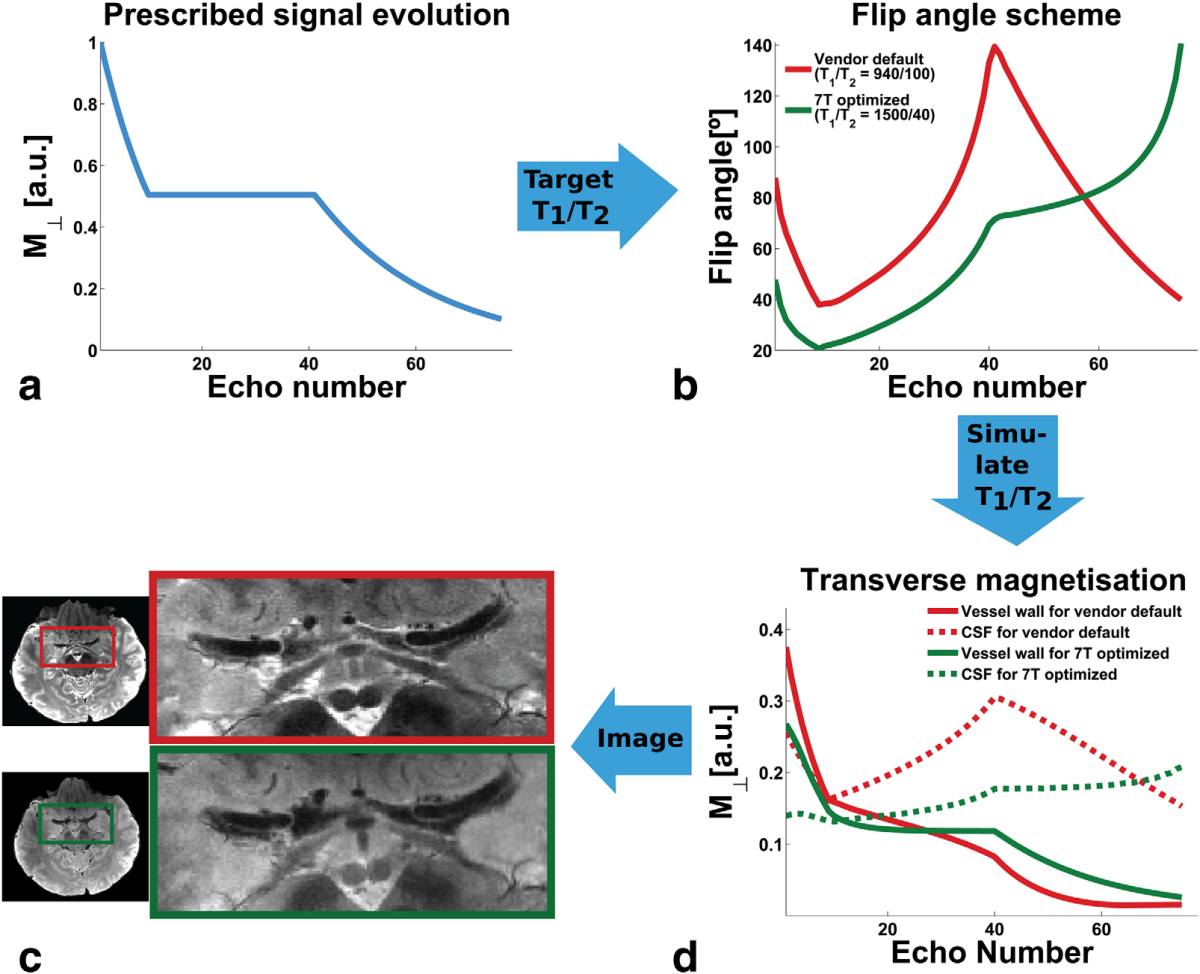
Schematic overview of the SPACE optimization. The prescribed signal evolution of the transverse component is shown in (a): The signal is kept constant over the central part of the readout (central part of k‐space for a T_2_‐weighted linear ascending readout scheme). The algorithm calculates the flip angles that produce the closest match to the prescribed signal for a given combination of T1 and T2 values (b). Red line: Flip angle scheme for the vendor‐implemented values of T_1_ = 940 ms and T_2_ = 100 ms. Green line: Flip angle scheme for vessel wall T_1_ = 1500 ms and T_2_ = 40 ms at 7T. MTF for vessel wall (solid lines) and CSF (dotted lines), simulated for both flip angle schemes (red for default, green for optimized) (c). Zoom of the MCA region for the same subject scanned with DANTE‐SPACE using the default scheme (red frame) and the optimized scheme (green frame) (d).

The magnetization transfer function (MTF) of a spin echo train is more distorted at higher field and leads to image blurring because of the faster $T_{2}$ decay. Flip angle sweeps, calculated with a Bloch formalism, are used to counteract this effect 14, 15. The goal is to find a scheme with a flat MTF over the central part of k‐space, assuming some default relaxation times $T_{1}$ and $T_{2}$. Figure 2a shows the principal steps of the optimization. The starting point is the vendor‐provided prescribed signal evolution for $T_{2}$‐weigthed TSEs. This evolution is a combination of an initial mono‐exponential decay (with a time constant of 17.5 ms and a duration of 11.9 ms per 100 ms of total echo train length (ETL)), a constant part and a final mono‐exponential decay (with a time constant of 29 ms and a duration of 46.5 ms per 100 ms ETL). It produces a narrower PSF than linear ramps or mono‐exponential decays (a comparison is provided in the Supporting Material, including Supporting Figure S1). To achieve a flat MTF, the flip angles need to be calculated that best match the evolution (Fig. 2b) for $T_{1}$ and $T_{2}$ values encountered by vessel wall at 7T. The calculated scheme can then be used to simulate the MTF for any tissue of interest, in our case vessel wall and CSF (Fig. 2c). The relative amplitudes of the MTFs indicate the contrast between static CSF and vessel wall. Because 7T measurements of intracranial vessel wall $T_{1}$ and $T_{2}$ were not available in the literature, we estimated values from carotid artery wall measurements at 3T, multiplying by scaling factors for white matter differences between 3T and 7T 16. We used factors of 1.45 17 and 0.67 18 for $T_{1}$ and $T_{2}$, respectively. Measured $T_{1}$ values of carotid arterial wall at 3T are approximately 1000 ms 19 and $T_{2}$ approximately 54 ms 20, resulting in $T_{1}$ and $T_{2}$ estimates of 1500 and 40 ms at 7T, respectively. This contrasted with the vendor‐implemented $T_{1}\;$ of 940 ms and $T_{2}\;$ of 100 ms. A flip angle scheme was therefore calculated for the estimated relaxation times. The Fourier transform of the MTF is the PSF along the phase encode direction. The full width at half maximum (FWHM) of the vessel wall PSF was calculated as an indicator of achievable image resolution for the vendor‐implemented scheme and the new scheme. Note that a 7T study by Koning et al 21 (published after our own study had commenced) reported carotid vessel wall $T_{1}=1628\;\pm 13\;$ ms and $T_{2}=46\;\pm 4$ ms, in agreement with our estimates within the standard deviation.

### DANTE Optimization

The goal of the DANTE optimization is to maximize the contrast between the outer wall and the surrounding CSF, ie, the difference in the longitudinal magnetizations, $M_{z},$ weighted by their spin densities $\;\rho_{0}$

(1) $$\Delta M_{z,VW/CSF}=\rho_{0,VW}\cdot M_{z,VW}-\rho_{0,CSF}\cdot |M_{z,CSF}|$$

The absolute value of the CSF signal is considered in Eq. (1), as oscillations into the steady‐state can lead to a negative undershoot of $M_{z,\;CSF}$ for a certain range of pulse numbers, whereas the MR image itself displays only the magnitude. The same equation applies to the inner wall and lumen contrast $\Delta M_{z,VW/Blood}.$ Contrast between moving and static spin isochromats in DANTE arises from a velocity‐induced phase spoiling effect that builds up with an increasing number of pulses, with the interpulse phase accumulation Δφ(n) given by

(2) $$\Delta \varphi \left(n\right)=\gamma \overset{n\tau_{G,D}}{\underset{\left(n-1\right)\tau_{G,D}}{\int }}\vec{G}\left(t\right)\cdot \vec{r}\left(t\right)dt\approx \underset{\;}{\underset{constant\;phase}{\underset{︸}{\gamma \vec{G}\vec{r_{0}}\tau_{G,D}}}}+\underset{}{\underset{}{\underset{velocity-induced\;phase\;spoiling}{\underset{︸}{\gamma \left(n-\frac{1}{2}\right)\vec{G}\vec{v}\tau_{G,D}^{2}}}}},$$

where $\vec{G}$ is the gradient, $\vec{r}$ the position, $\vec{v}$ the velocity vector, and $\tau_{G,D}$ the gradient duration. A detailed mathematical description of the signal evolution during DANTE can be found in 10. The major uncertainty in simulations is the CSF velocity in the MCA vicinity. The predominant pattern for CSF velocity fluctuations is sinusoidal with the cardiac cycle. The highest amplitudes have been measured to be in the range of 3–5 cm/s in dynamic flow regions such as the cerebral aqueduct 22, 23. In many other areas, such as the subarachnoid space, CSF is almost static and the DANTE‐spoiling effect is expected to be negligible. Eq. (2) suggests that larger gradient amplitudes lead to a more efficient phase spoiling, although peripheral nerve stimulation imposes an upper limit. Decreasing the slew rate at the cost of longer gradient durations $\tau_{G,D}$ is not a viable option, as contrast decreases with longer pulse‐to‐pulse durations 12. We simulated the magnetization of vessel wall, dynamic CSF, and blood voxels using MATLAB (Mathworks, Natick, Massachusetts). Simulations were run for a flip angle range of 7–12 ° and up to 700 pulses. In addition to exploring the range of suitable parameters, this flip angle range further addresses the B_1_ variations, which are increased at 7T. A CSF or blood voxel consists of multiple spin isochromats of different velocities. This velocity dispersion was taken into account by averaging over a range of velocities. A CSF voxel was modeled to have velocities of 2 cm/s ± 2.5% in the *z* direction and 0.5 cm/s ± 2.5% in the other directions. The blood velocities were set to 20 cm/s ± 2.5% in the *z* direction as measured in the carotid artery 24. All further simulation parameters were chosen to match the optimal sequence parameters achievable by the scanner and were in accordance with nerve stimulation limits. We chose the maximum gradient amplitude along the head–feet direction, as this is the major direction of CSF and blood flow: G_A–P_ = 28 mT/m, G_R–L_ = 20 mT/m, G_H–F_ = 40 mT/m. An interpulse time $\tau_{D}$ of 1.6 ms was used, which is at the limit of our scanner's mechanical resonance response. Other parameters were $\tau_{G,D}=1.4$ ms, $T_{1,CSF}/T_{2,CSF}=4019/311$ ms (average values from 17, 25), $T_{1,Blood}/T_{2,Blood}=2290/100\;\text{ms}$ (for arterial blood 26) and a voxel size of $0.5\times 0.5\times 1\;{\text{mm}}^{3}$. Vessel wall relaxation times were $T_{1,VW}/T_{2,VW}=1500/40$ ms. To calculate the contrasts $\Delta M_{z,VW/CSF}\;$ and $\Delta M_{z,VW/Blood}$ from Eq. (1), we used a normalized CSF and blood spin density of 1 and assumed a vessel wall spin density of 0.72 (average of gray and white matter spin densities relative to CSF 27). A common simplification in DANTE simulations of tissue is the assumption of no movement. However, brain movement is a known phenomenon 28, 29, and a detailed study of potential signal loss during the DANTE preparation is provided in the Supporting Material (Supporting Table S2).

### MR Imaging

All scans were run on a 7T scanner (Siemens Magnetom, Erlangen, Germany) using a 32‐channel head coil (Nova Medical, Wilmington, Massachusetts) and under institutional ethical approval.

A SPACE scan with the vendor‐implemented scheme and the optimized scheme were acquired in four subjects (mean age = 32) for a comparison of wall depiction. DANTE was applied for these scans to reduce the CSF signal as much as possible to enhance vessel wall conspicuity for line profile measurements (see “Image Analysis” section).

To test the effect of DANTE, we ran the 7T‐optimized SPACE sequence with and without DANTE in 11 healthy volunteers (four females, age range 25–64 years, mean age = 35). For all scans, the following settings apply: DANTE: FA = 10 °, n = 300, $\tau_{D}$ = 1.6 ms, $\tau_{G,D}\;$ = 1.4 ms; SPACE: transversal orientation, voxel size = $\;0.5\times 0.5\times 1{\;\text{mm}}^{3}$, field of view (FOV_read_) = 240 mm, FOV_phase_ = 75%, 176 slices, TR/TE_equivalent_ = 2620/165 ms, echo spacing = 4.6 ms, ETL = 345 ms, GRAPPA = 4 in the phase‐encode direction, turbo factor = 39, slice turbo factor = 2, echo trains per slice = 3, BW = 465 Hz/pixel. The total scan time was approximately 11 min for each scan.

For SNR measurements (see “Image Analysis” section) we had to calculate the spatially varying noise amplification resulting from the parallel acceleration used in the sequences. For this purpose we acquired a fully sampled GRE scan with and without RF pulses (1 minute duration each) 30 in four subjects. The same FOV as in the DANTE‐SPACE and SPACE scans was used. The raw k‐space data were loaded offline.

### Image Analysis of Vessel Wall Conspicuity: SPACE Optimization

**Figure 3:**
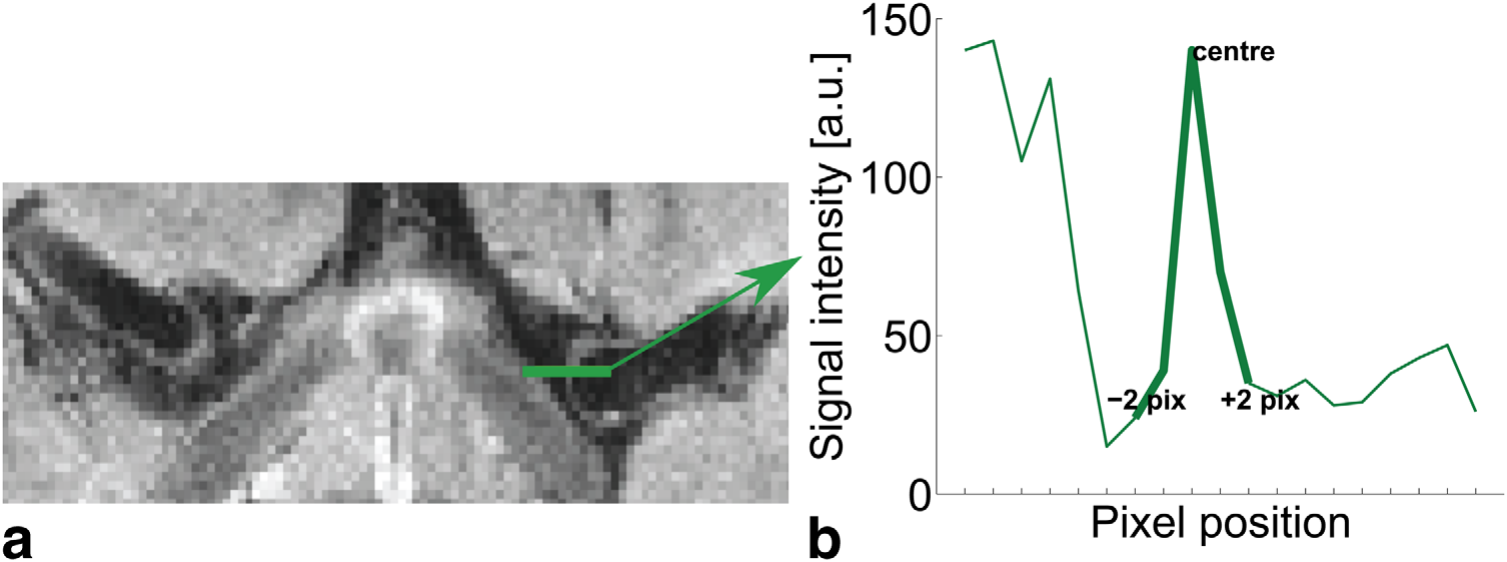
Example intensity line profile. The profile (green line) is positioned parallel to the phase‐encode direction in a location where the wall is perpendicular to that direction (a); the intensity profile at that location is shown in (b). The values used for the Gaussian fit are highlighted in bold.

Intensity line profiles across the MCA wall were positioned parallel to the phase‐encode direction (R–L) in locations where the wall was aligned perpendicularly and was not touching surrounding tissue (Fig. 3a). To increase the number of profiles that meet both conditions, we further included profiles across the basilar artery. Across all four subjects, a total of 24 profiles were identified. The profile maximum was assigned the PSF center. Two pixels to each side from the center (Fig. 3b) were included in the fitting of a Gaussian profile. The FWHM was calculated from the fitted parameters. To compare the CSF signal under both schemes, we further measured four CSF regions of interest (ROI) (two in the ventricles and two in the subarachnoid spaces) in each subject. Similarly, the vessel wall signal changes were assessed using the PSF center value. All analyses were carried out in MATLAB.

### Image Analysis of Vessel Wall Boundary Contrast: DANTE‐SPACE Versus SPACE

**Figure 4:**
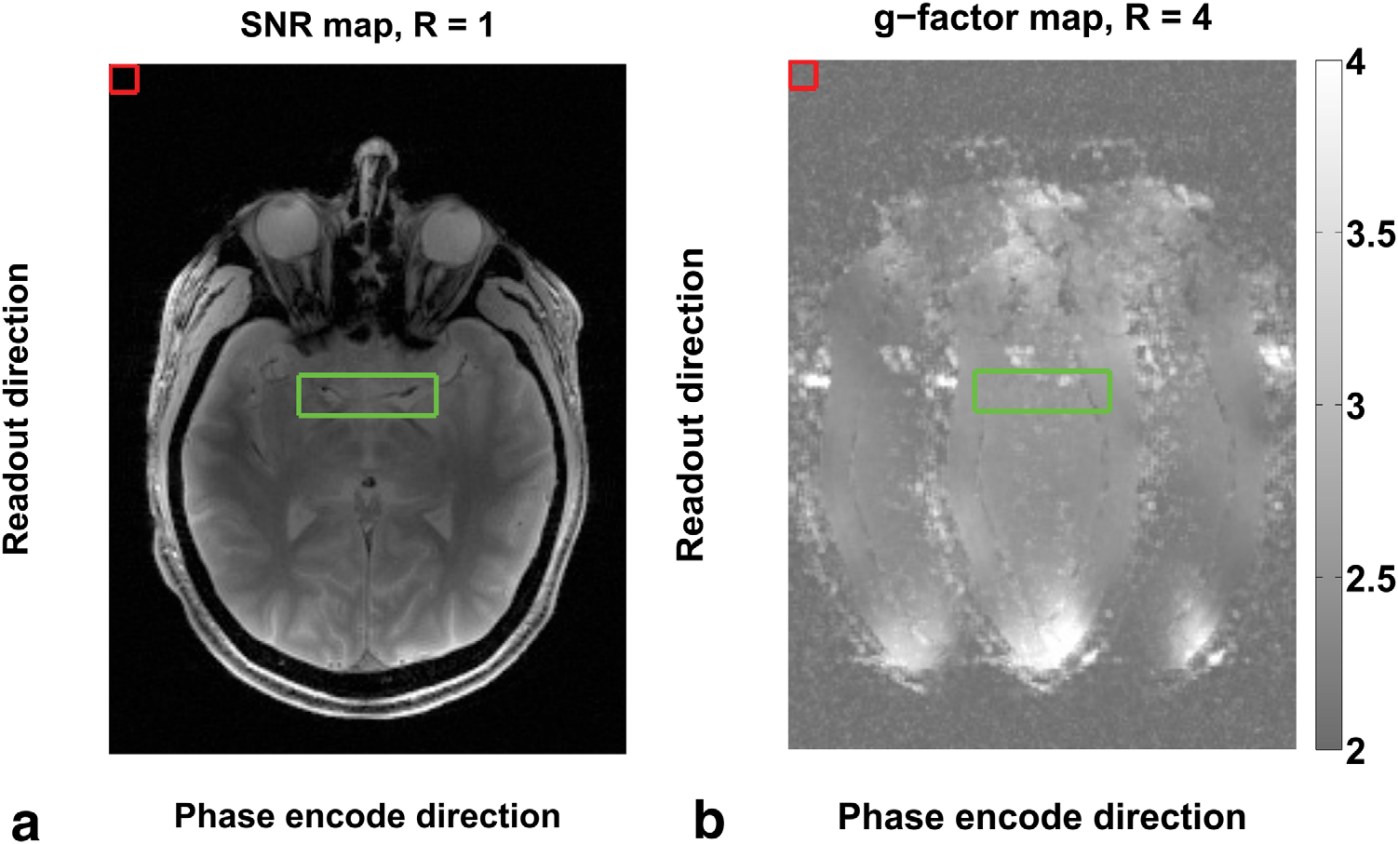
Nonaccelerated SNR map with the MCA (green box) and noise area (red box) (a). g‐Factor map for parallel acceleration in the phase‐encode direction of factor 4 (b). The ratio of the mean g‐factors within the ROIs is used to scale the thermal noise in the parallel‐accelerated DANTE‐SPACE and SPACE scans.

To account for the spatially varying noise amplification from the GRAPPA acceleration, we defined SNR as

(3) $$SNR_{tissue}=\frac{S_{tissue}}{G\cdot \sigma_{noise}},$$

where $S_{tissue}$ is the signal in the tissue mask and $\sigma_{noise}$ is the standard deviation in a noise mask in a ghost‐free area outside of the brain and

(4) $$G=\frac{g_{MCA\;ROI}}{g_{noise\;ROI}}$$

is the ratio of the g‐factors in the noise and tissue areas. Thermal noise cannot be measured reliably in tissue areas itself, but multiplying $\sigma_{noise}$ by G accounts for the amplified noise in the MCA area. To calculate the g‐factor maps, SNR maps were simulated in MATLAB with the “pseudo multiple replica” method by Robson et al 30. This method uses the nonaccelerated RF‐free k‐space data to derive the noise covariance matrix to account for spatial correlations and scaling. Corrected artificial noise is then added to the k‐space data to form multiple replicas of the true data that only vary by noise. Images are reconstructed for each replica. For each pixel the magnitude in the true reconstruction is then divided by the standard deviation over all replica reconstructions to yield the SNR map. For each subject, a region over multiple slices that contained the MCA was identified on the nonaccelerated SNR map and transferred to the g‐factor map to calculate the local $g_{MCA\;ROI}$. A noise area was selected in the upper‐right corner of the FOV to yield $g_{noise\;ROI}$. Example SNR and g‐factor maps are provided in Figure 4, showing the areas that were used to calculate G. An average G value was calculated from the four subjects, and applied to all subsequent analyses.

**Figure 5:**
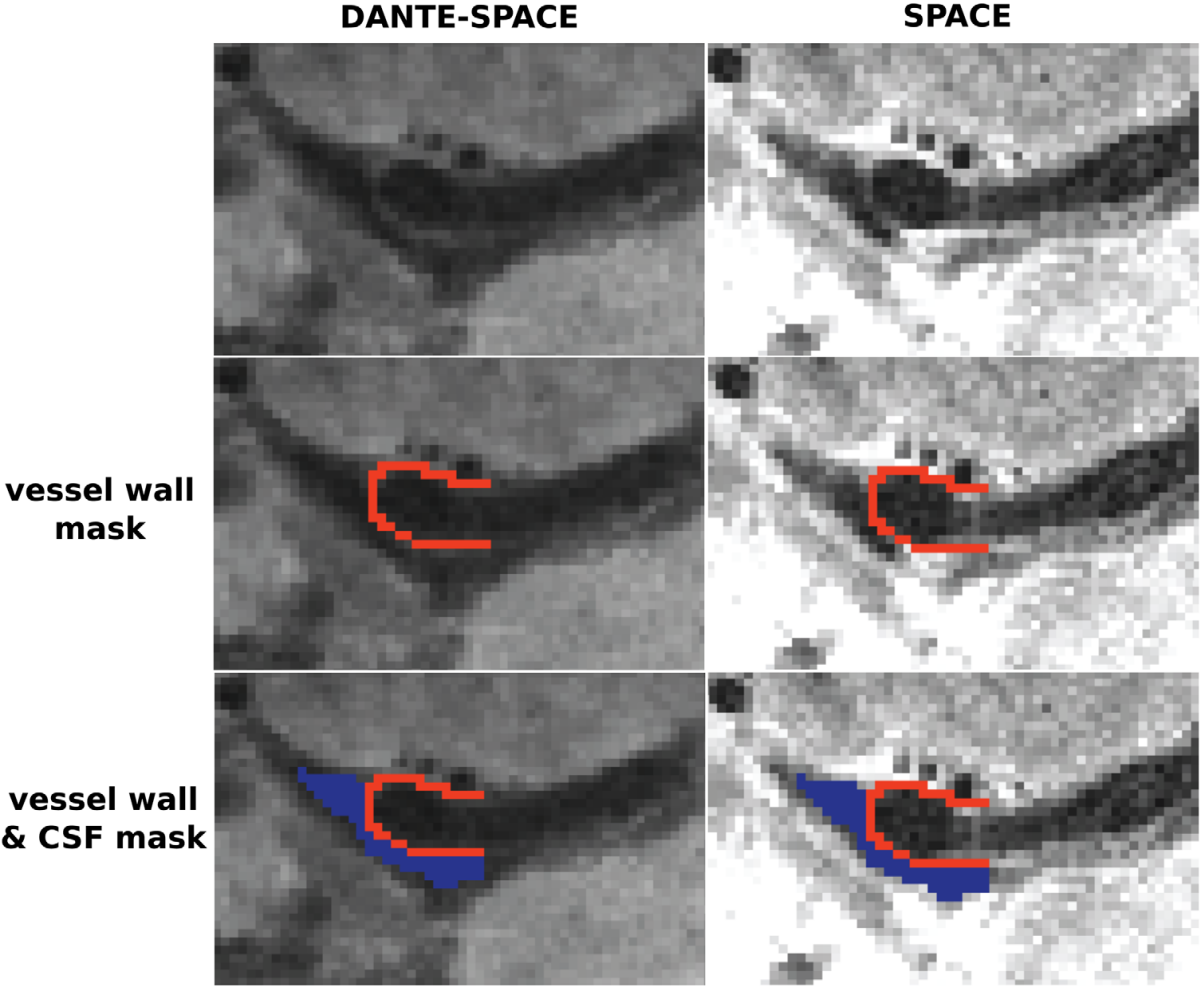
Location of the signal masks. The vessel wall mask (red) and CSF mask (blue) were drawn first on the DANTE‐SPACE scan (left column) and then applied to the registered SPACE scan (right column).

To assess the ability of DANTE‐SPACE to improve outer wall contrast, we measured the CNR between wall and surrounding CSF, defined as $CNR_{VW/CSF}=\;SNR_{VW}-SNR_{CSF}$. $CNR_{VW/Lumen}$ between wall and lumen was measured similarly. Additionally, we calculated the signal ratios between wall and CSF as $SR_{VW/CSF}=\frac{S_{VW}}{S_{CSF}}$ and $SR_{VW/Lumen}\;\;$ between wall and lumen, respectively. We drew two ROIs on each side of the MCA within each subject (total of four ROIs per subject) using FSLview (FMRIB Software Library). We used the DANTE‐SPACE rather than the SPACE scan, as it was more difficult to distinguish between wall, tissue, and CSF signal on the latter because of the higher CSF signal. Both scans were registered linearly within each subject using the FSL tool FLIRT 31. We checked that registration was successful for every subject to avoid any bias when transforming the ROI masks. Figure 5 shows an example of the ROI selection process. A student's paired‐sample t‐test was used for statistical analysis.

### Patient Scan

To test the ability of DANTE‐SPACE to depict pathology, we scanned a 31‐year‐old female patient diagnosed with a lacunar infarct in the right hemisphere and stenosis in the right MCA M1 segment. We ran a T1‐MPRAGE in that subject to visualize the blood flow and a DANTE‐SPACE scan for VWI (settings as described previously). The T1‐MPRAGE settings were as follows: voxel = $0.8\times 0.8\times 0.8mm^{3}$, FOV_read_ = 256 mm, FOV_phase_ = 100%, 256 slices, TR = 2200 ms, TE = 2.9 ms, TI = 1050 ms, FA = 7 °, GRAPPA = 2, BW = 240 Hz/px, echo spacing = 7.2 ms. Scan time was 7 min.

## RESULTS

### SPACE Optimization

Figure 2a shows the prescribed MTF used for the flip angle calculation. Figure 2b plots the flip angle schemes that arise from the default implementation (red) and the calculation based on vessel wall relaxation times (green). As an appreciated by‐product, the new scheme reduces the overall flip angle amplitudes (red versus green lines in Fig. 2b) and therefore SAR, allowing for shorter TR and scan time. The flip angles for the optimized scheme are provided in the Supporting Materials. The MTFs of the schemes are plotted in Figure 2c for vessel wall (solid lines) and CSF (dotted lines). (Note that Fig. 2c shows the predicted signal of static CSF with no motion‐induced effects taken into account, which will suppress the CSF signal further.) The FWHM of the theoretically derived vessel wall PSF and the FWHM of the experimentally measured line profiles are provided in Table 1. The theoretical FWHM of the default implementation is 1.73 pixels versus 1.29 pixels for the optimized implementation, predicting an improvement in vessel wall resolution of 25%. The experimentally measured FWHM reduction was 17%. Example images with (green) and without (red) optimization are shown in Figure 2d. The MTF simulations predict a reduction of the CSF $M_{⊥}$ component (dotted red versus dotted green line in Fig. 2c), whereas the vessel wall $M_{⊥}$ component is almost unchanged (solid red versus solid green line). From the ROI measurements we found a CSF signal reduction of 38% from the default to optimized scheme, but also a reduction of vessel wall signal of 33%, which is not expected from the MTF simulations.

**Table 1:** PSF Results for the Default and Optimized Flip Angle Schemes^a^

Method	Default scheme	Optimized scheme
FWHM of PSF derived from Bloch simulations	1.73	1.29
FWHM ± σ from Gaussian fit to vessel wall line profiles	3.38 ± 0.76	2.88 ± 0.95

a Values are in the units of pixels.

### DANTE Optimization

**Figure 6:**
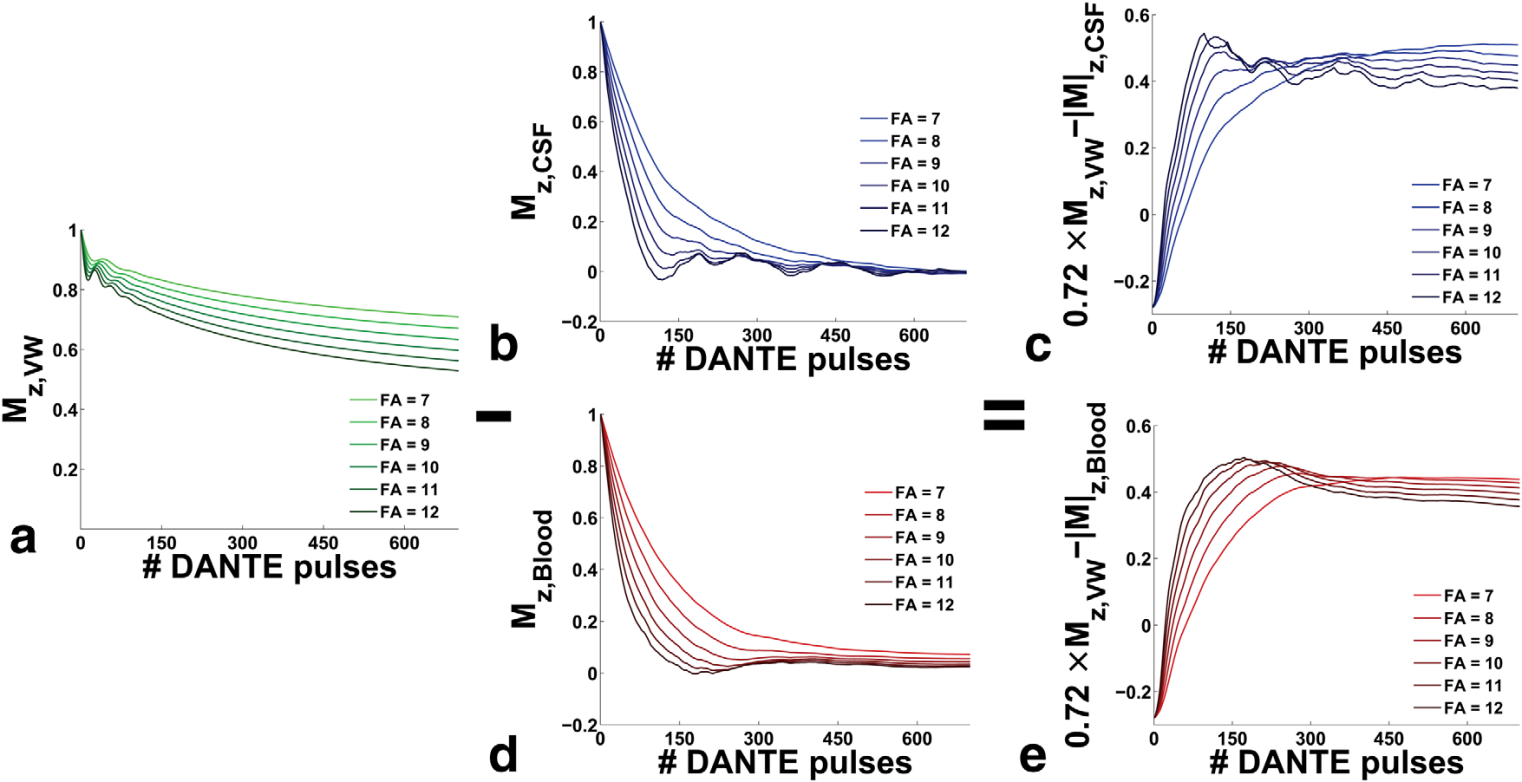
Simulation results of the longitudinal magnetization of vessel wall, flowing CSF, and arterial blood for an increasing number of DANTE pulses. Results are plotted for a range of flip angles from 7° (light color) to 12° (dark color). DANTE steady‐state approach for vessel wall tissue (a). Signal crushing effect of DANTE for a CSF voxel (b). Resulting contrast between vessel wall and CSF (c). Signal crushing effect of DANTE for a blood voxel (d). Resulting contrast between vessel wall and blood (e).

Figure 6a shows the simulated approach to steady state of the $M_{z,VW}$ component as a function of increasing flip angle and number of DANTE pulses. Figures 6b and 6d show the predicted spoiling of $M_{z,CSF}$ and $M_{z,Blood}$. The achievable crushing effect scales with flip angle, with almost complete spoiling for 10 ° and 150 pulses, whereas this level is only reached with 500 pulses when the flip angle is 7 °, suggesting a significant variation with actual flip angle and B_1_ inhomogeneity. Figures 6c and 6d show the resulting contrast between wall and CSF, and between wall and blood, respectively. Contrast is predicted to peak earlier for higher flip angles, but with an overall lower steady state compared with smaller flip angles. Empirically, on the scanner we found insufficient CSF signal crushing effect for a short pulse train and a high flip angle of 12 ° and had to increase the number of pulses to 300 with a flip angle of 10 °, suggesting B_1_ inhomogeneities and flip angle drop‐off towards the neck. A lower flip angle with more preparation pulses is preferred over a larger flip angle with fewer pulses, as the latter will decrease the tissue steady‐state signal in regions where B_1_ is accurate.

### MR Imaging: DANTE‐SPACE Versus SPACE

**Figure 7:**
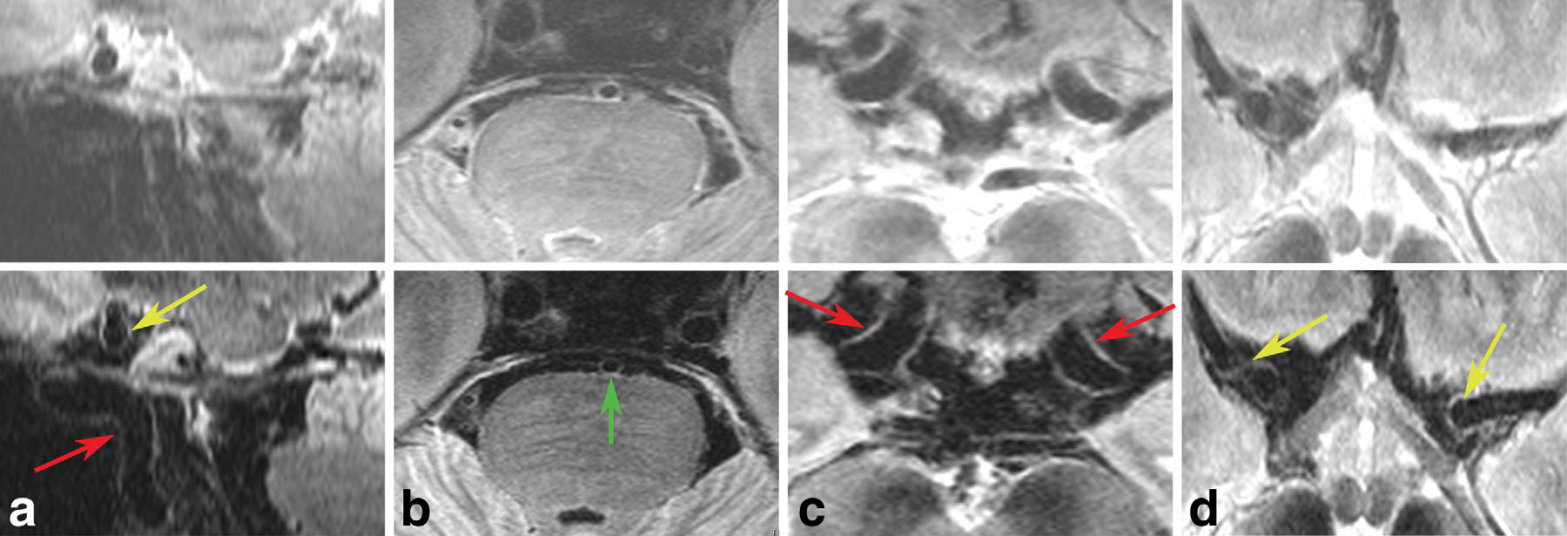
SPACE (top row) and DANTE‐SPACE (bottom row) scans of a 64‐year‐old male subject with no known neuropathological condition. Both scans are shown at the same signal level, but the SPACE scans are displayed at double the signal width. Coronal view of the internal carotid artery (red arrow) and the cross section of the MCA (yellow arrow) (a). Sagittal view of the brainstem with the basilar artery (green arrow) (b). Sagittal view of the internal carotid arteries (red arrows) at the level where they split into the posterior communicating artery and the MCA (c). M1 segments of the right and left MCA (yellow arrows) (d).

The g‐factor measurements gave a mean scaling factor G of 1.27 with a standard deviation of 0.05 ( < 4%) between subjects. The results of the SNR, SR, and CNR measurements are provided in Table 2 (in two subjects we could only identify one ROI on the right side, as the MCA wall was adjacent to the cortex and not surrounded by CSF, yielding a total of 42 ROIs). $SNR_{VW}\;\;$ drops by almost a factor of two for the DANTE preparation. However, as the decrease of $SNR_{CSF}$ is larger, there is an improvement in outer wall boundary contrast $CNR_{VW/CSF}$ for DANTE‐SPACE compared with SPACE. This improvement was significant (*P* = 0.0159) in the right hemisphere, but not in the left (*P* = 0.09). Generally, values vary strongly, with stronger variations for the SPACE scans without DANTE. The $\;\;CNR_{VW/CSF}$ variations occur within and across subjects. Thirty‐two out of the 42 ROIs ( ≈ 76%) have positive $CNR_{VW/CSF}$ on the SPACE scans, which increases to 40 ROIs ( ≈ 95%) with positive contrast on DANTE‐SPACE scans. $SNR_{Lumen}$ in the lumen area decreases on DANTE‐SPACE scans, but the reduction is not statistically significant (*P* = 0.089 for the right and *P* = 0.055 for the left). In combination with the $SNR_{VW}$ reduction, this leads to a decrease of inner wall boundary contrast $CNR_{VW/Lumen}$. $SR_{VW/CSF}$ increases significantly for the DANTE‐prepared scans in both hemispheres (*P* < 10^−4^ for the right and *P* < 10^−5^ for the left). $SR_{VW/Lumen}$ decreases significantly for DANTE‐SPACE, again because of the decrease in vessel wall signal. Sample DANTE‐SPACE and SPACE images are shown in Figure 7 for a 64‐year‐old healthy subject.

**Table 2:** SNR, SR, and CNR Results^a^

Compartment			DANTE	No DANTE	*P* value
SNR ±σ	Vessel wall	right	43.01 ± 20.86	71.67 ± 48.01	<10^−3^
		left	40.12 ± 15.99	80.0 ± 51.98	<10^−4^
	CSF	right	30.66 ± 17.93	66.06 ± 51.64	<10^−3^
		left	28.23 ± 15.22	74.47 ± 63.56	<10^−3^
	Lumen	right	15.96 ± 5.7	18.13 ± 9.38	0.09
		left	17.32 ± 6.38	20.53 ± 11.69	0.055
SR ±σ	Vessel wall versus CSF	right	1.47 ± 0.28	1.22 ± 0.25	<10^−4^
		left	1.44 ± 0.37	1.18 ± 0.35	<10^−5^
	Vessel wall versus Lumen	right	2.62 ± 0.45	3.9 ± 1.06	<10^−6^
		left	2.32 ± 0.32	3.93 ± 0.87	<10^−8^
CNR ±σ	Vessel wall versus CSF	right	12.35 ± 8.51	5.62 ± 11.17	0.0159
		left	11.89 ± 6.22	5.53 ± 19.35	0.09
	Vessel wall versus Lumen	right	27.04 ± 15.75	53.54 ± 40.35	<10^−3^
		left	22.81 ± 10.39	59.47 ± 41.78	<10^−4^

a A total of 42 ROIs from 11 subjects (four ROIs per subject; for two subjects only three ROIs could be defined). Mean values and standard deviations are listed for the DANTE‐SPACE and the SPACE scan. The *P* value corresponds to a paired t‐test.

### Patient Scan

**Figure 8:**
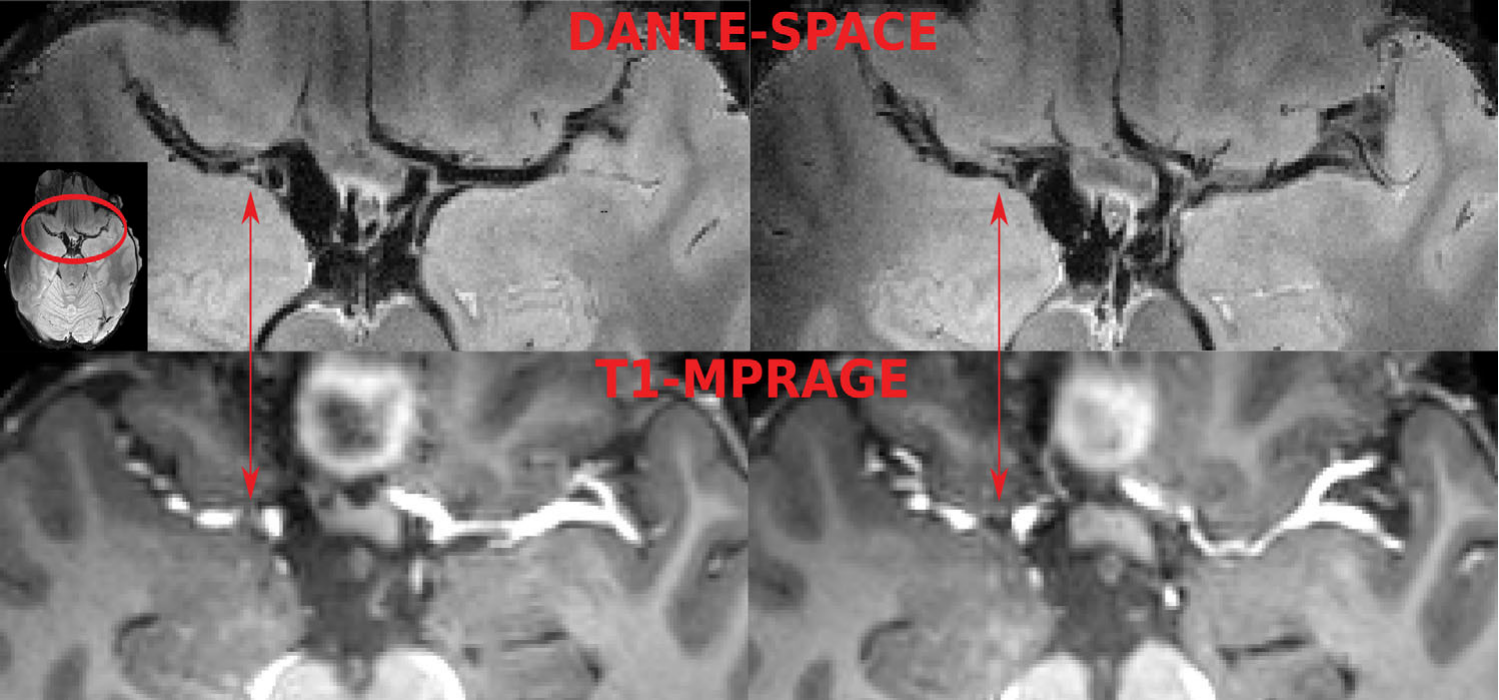
Two sample images in a lacunar stroke patient with stenosis in the right M1 segment. The red arrows indicate the side of stenosis on the DANTE‐SPACE vessel wall scans (top row) and the T1‐MPRAGE bright‐blood scans (bottom row).

Figure 8 shows two slices from the exemplar stenosis patient. The regions of hindered blood flow, visible on the bright‐blood T1‐MPRAGE scan, coincide with the vessel wall structure on the DANTE‐SPACE scan (red arrows).

## DISCUSSION

### Vessel Wall Conspicuity: SPACE Optimization

The proposed optimized variable flip angle scheme produced a sharper vessel wall PSF, reduced CSF signal, and reduced SAR compared with the vendor‐provided implementation. The improvement in vessel wall PSF was theoretically predicted to be 25%, and experimentally a narrowing of the FWHM of 17% was measured. However, the measured FWHM was approximately double the theoretical prediction: 2.88 versus 1.29 pixels and 3.38 versus 1.73 pixels, for the optimized and default schemes, respectively. This is likely the result of inaccuracies in the measurement and fitting of the line profiles. We cannot assume that the wall was a point‐like structure along the profile. As the wall structure is assumed to be smaller than the image resolution, CSF and blood at the boundary produce a partial volume effect. These factors further explain the PSF broadening and are likely to produce the vessel wall signal reduction that is observed in the measurement but is not predicted by the MTF simulation, which assumes pure vessel wall tissue.

### Vessel Wall Boundary Contrast: DANTE‐SPACE Versus SPACE

To further reduce the CSF signal for outer wall contrast enhancement, a DANTE preparation was applied. There is a mismatch between optimal DANTE parameters derived from the simulation and experimental findings. One explanation is the inhomogeneous B_1_ field and flip angles chosen in the protocol are likely not achieved in the lower brain regions because of the poorer excitation profile towards the neck for 7T head coils. A large variation in $SNR_{CSF}$ and $CNR_{VW/CSF}$ was found across and within subjects and is likely the result of variations in CSF dynamics. $CNR_{VW/CSF}$ measurements showed an improvement for DANTE‐SPACE versus SPACE in the MCA, but this was only statistically significant in the right hemisphere. Generally, $CNR_{VW/CSF}\;$ values were found to be lower in the left hemisphere, likely to be caused by the asymmetric right–left excitation profile for T2‐SPACE acquisitions, with reduced contrast in the left hemisphere 32. Parallel transmit approaches may help to address this for both methods. The signal ratio between wall and CSF is increased and the contrast is doubled in DANTE‐SPACE, enhancing the outer wall boundary contrast. This comes with the tradeoff of reduced inner boundary contrast, which is halved, but still at a high level of approximately 25. Sufficient signal ratio between both boundaries is crucial for plaque assessment.

The major limitation of this study is the resolution ( $0.5\times 0.5\times 1\;{\text{mm}}^{3}$). The MCA wall thickness is assumed to be in the range of 0.2 mm for healthy subjects. In line with the vessel wall PSF measurements, we found a larger $SNR_{VW}$ drop than expected from the simulations, and the same partial volume effect argument holds. However, increasing the resolution is not straightforward, because of the scan time, DANTE banding patterns, and SAR. Nevertheless, wall thickening with age could make DANTE‐SPACE the preferred VWI sequence over SPACE. The images of the stenosis patient show the potential of DANTE‐SPACE to depict vessel wall architecture and pathology. However, ultimately a study with a large number of patients with pathology would be needed.

A recent review of the literature on VWI (35) lists only magnetization‐prepared inversion recovery TSE (MPIR‐TSE) 6, 34 as an alternative sequence for VWI in vivo at 7T. That sequence has an acquisition time of 11–12 min, comparable to our protocol. The MPIR‐TSE sequence has been used with a lower, but isotropic, resolution of 0.8 × 0.8 × 0.8 mm^3^. We chose a conservatively long TR to guarantee SAR compliance for every head geometry and to keep the imaging protocol consistent (SAR ranged between subjects from 66 to 98%). In a clinical setting the TR could be adjusted individually to reduce the scan time. Here, we present only T_2_‐weighted images; future work should address the implementation of multicontrast DANTE‐SPACE at 7T. Other possible improvements could be an increase in resolution, trading off the size of the imaging slab, similar to Qiao et al 8, who presented high‐resolution VWI of up to 0.4 mm at 3T with the VISTA sequence.

## CONCLUSION

The optimization of the SPACE readout produces a sharper vessel wall depiction and reduces SAR at 7T. DANTE‐SPACE doubles the contrast between wall and CSF compared with SPACE only, which comes with the tradeoff of lower wall‐to‐lumen contrast that can be tolerated, given that this contrast is generally higher than the contrast to the outer boundary. Signal level and contrast show a strong inter‐ and intrasubject variability, which is a weakness of velocity‐induced contrast techniques such as DANTE‐SPACE over methods that use inversion to create contrast.

## Supporting information

Additional Supporting Information may be found in the online version of this article

**Fig. S1.** Comparison of three different signal evolution schemes and their PSF. Signal evolution for a linear ramp (green line), a mono‐exponential decay (red line), and a combination of an initial exponential decay, followed by a flat period, finishing with an exponential decay (blue line) (a). MTFs of the vessel wall tissue for the three evolution schemes (b). The PSFs of the MTFs and their FWHM (c).
**Table S2. R**esults of the Brain Motion‐Induced Signal Loss Measurements Using Single Directional DANTE Gradients^a^
^a^The numbers are the mean signal intensity ratios ( ± standard deviation) between the acquisitions with and without applied DANTE gradient for the four subjects. Signal loss varies for different DANTE directions when measured in the human brains, but is directionally independent of the rigid pork phantom.
