# Supplementary material for: T2‐Weighted intracranial vessel wall imaging at 7 Tesla using a DANTE‐prepared variable flip angle turbo spin echo readout (DANTE‐SPACE)
Source: Magn Reson Med. 2016 Feb 18;77(2):655–63. doi: 10.1002/mrm.26152 (PMC5298018; doi:10.1002/mrm.26152)
Supplement: Supplementary file 1 — Fig. S1. Comparison of three different signal evolution schemes and their PSF. Signal evolution for a linear ramp (green line), a mono‐exponential decay (red line), and a combination of an initial exponential decay, followed by a flat period, finishing with an exponential decay (blue line) (a). MTFs of the vessel wall tissue for the three evolution schemes (b). The PSFs of the MTFs and their FWHM (c). Table S2. Results of the Brain Motion‐Induced Signal Loss Measurements Using Single Directional DANTE Gradientsa aThe numbers are the mean signal intensity ratios ( ± standard deviation) between the acquisitions with and without applied DANTE gradient for the four subjects. Signal loss varies for different DANTE directions when measured in the human brains, but is directionally independent of the rigid pork phantom. [file MRM-77-655-s001.docx]

**Supporting Material**

**SPACE Optimization – Comparison of Evolution Schemes**

Supporting Fig. S1a) shows three different signal evolution schemes: A combination of an exponential decay, a flat part and a final exponential decay (blue), a linear ramp (green) and a mono-exponential decay (red). The realised MTFs for vessel wall for these schemes are shown in b) and the resulting PSF and FWHMs are given in c). The PSF for the exponential-flat-exponential scheme is narrower than a linear ramp or a mono-exponential decayWires.

**Supporting figure S1:**

**SPACE Optimization – Flip Angle Scheme**

The optimised flip angle scheme consists of the following flip angles (in degrees): 90.0 86.9 47.7 37.7 31.9 28.9 26.4 24.7 23.1 21.8 20.6 21.6 22.1 22.8 23.3 24.1 24.8 25.6 26.4 27.4 28.3 29.3 30.3 31.4 32.5 33.6 34.8 36.0 37.3 38.8 40.2 41.8 43.6 45.4 47.4 49.6 52.0 54.6 57.6 60.9 64.8 69.2 71.5 72.6 73.1 73.3 73.7 74.1 74.6 75.0 75.5 75.9 76.5 77.0 77.6 78.1 78.8 79.5 80.2 81.0 81.9 82.9 83.9 85.1 86.3 87.8 89.4 91.3 93.4 95.8 98.7 102.0 106.1 111.2 117.8 126.7 140.6

**Signal loss due to brain movement**

We investigated the level of signal loss in static brain tissue caused by the DANTE module beyond the expected value. We anticipated potential signal loss in the region of the brain stem, which is known to move throughout the cardiac cycle, and which lies close to the MCA. The brain and the intracranial vasculature move with the cardiac cycle. This movement is anisotropic and peak amplitudes of 1.5 - 2mm/s in a caudally-anterior direction have been measured in the brainstem (Wirestam et al. JMRI, 1996; Greitz et al., Neuroradiology, 1992). A potential concern is that the strong gradients (up to 40mT/m in this work) used in DANTE may attenuate signal from ‘static’ tissue as well. When single directional gradients along the principal axes are applied, i.e. $\vec{G}=G\hat{e_{i}}$, the velocity-induced phase term in Eqn. 2 (main manuscript) will solely depend on the velocity amplitude along that direction, i.e. $Gv_{i}$. If DANTE preparation causes additional signal loss in tissue due to anisotropic brain movement, the image intensity should vary between DANTE scans that are run with different single directional gradients. Indeed, the observations above would predict that a DANTE scan acquired with a gradient along the inferior-superior direction leads to more signal dropout in the brain stem compared to a DANTE scan acquired with a gradient along the right-left direction.

*MR Imaging*

To assess the effect of brain motion on DANTE-induced static signal attenuation we used DANTE preparations with the gradient aligned along each of the three principal axes in turn: head>>feet $G_{H\gg F}$, anterior>>posterior $G_{A\gg P}$and right>>left $G_{R\gg L}$. We acquired data in four healthy volunteers and in a pork phantom. The pork phantom served as a truly rigid tissue control for validation. Each subject underwent a total of four scans, consisting of the three single-directional DANTE-SPACE scans and a SPACE scan with DANTE switched off for normalisation.

*Imaging parameters*

*DANTE:* Parameters were the same as for the DANTE preparation described in the main manuscript, with the exception that the gradients were only switched on along a single axis at the maximum amplitude of $G=40mT/m$. *SPACE:* voxel = $1\times1\times1{mm}^{3}$, FOV_read_=220mm, FOV_phase_=75%, base resolution=258, 176 slices, TR/TE_equivalent_=3980/198ms, inter-echo spacing=3.4ms, ETL=401ms, GRAPPA=4, BW=592 Hz/pixel.

*Image analysis*

To ensure that the signal is evaluated in equivalent regions across the subjects all datasets were non-linearly registered to a $T_{2}$ template using FSL. Three ROIs were defined: one in the brain stem and one in the grey/white matter tissue proximal to the MCA in each hemisphere. The signal loss in each ROI was defined as the mean voxel signal ratio $\langle S_{DANTE-SPACE}/S_{SPACE} \rangle$, which was calculated for each of the three single directional DANTE gradient scans. The values were then averaged over all four subjects. The ROI in the pork phantom was drawn manually in a central part of the tissue.

*Results*

Results showing the effect of DANTE on static tissue are shown in the supplementary table S2, below. The pork phantom measurement confirms that there is no significant difference in signal reduction for different gradient directions in case of truly static tissue. However, in the brain stem ROI we measured an additional reduction along the head>>feet direction of 25% compared to the other two directions. The anterior>>posterior and right>>left directions do not differ significantly. In ROIs proximal to the MCA the signal ratios in the left hemisphere are lower for all ROIs compared to their right counterpart. Again, the head>>feet direction generates a higher signal loss than the other directions with a maximum of 11% compared to the right>>left direction.

Supporting table S2:

|  | | $\boldsymbol{\langle}\boldsymbol{S}_{\boldsymbol{DANTE-SPACE}}\boldsymbol{/}\boldsymbol{S}_{\boldsymbol{SPACE}} \rangle$ ± $\sigma$ | | |
| --- | --- | --- | --- | --- |
|  | | $G_{R\gg L}$  $=40mT/m\cdot\hat{e_{x}}$ | $G_{A\gg P}$  $=40mT/m\cdot\hat{e_{y}}$ | $G_{H\gg F}$  $=40mT/m\cdot\hat{e_{z}}$ |
| brainstem |  | 0.63 ± 0.02 | 0.64 ±0.05 | 0.47 ± 0.07 |
| close to right MCA | | 0.75 ± 0.03 | 0.71 ± 0.04 | 0.69 ± 0.02 |
| close to left MCA | | 0.72 ± 0.02 | 0.67 ± 0.01 | 0.64 ± 0.05 |
| static phantom | | 0.65 | 0.67 | 0.65 |

*Discussion*

The results suggest that there is additional signal loss in tissue close to the MCA region due to brain movement during DANTE-SPACE, with a worst-case loss of 13% in the head>>feet direction on the left hemisphere compared to the right>>left direction. However, this finding cannot be applied to the MCA vessel wall directly as there is additional pulsatility. This further underlines that DANTE induces unwanted signal reduction if there is sufficient movement in the direction of the DANTE gradients. Brain movement is thought to support the pumping of CSF through the ventricular system, hence CSF flow and brain movement are likely to be along similar directions, making the choice of an optimal DANTE gradient problematic.
